# White Matter Microstructural Damage Associated With Gait Abnormalities in Idiopathic Normal Pressure Hydrocephalus

**DOI:** 10.3389/fnagi.2021.660621

**Published:** 2021-08-09

**Authors:** Yan-min Tang, Ye Yao, Shuai Xu, Xin Li, Fan Hu, He Wang, Jing Ding, Xin Wang

**Affiliations:** ^1^Department of Neurology, Zhongshan Hospital, Fudan University, Shanghai, China; ^2^Department of Biostatistics, School of Public Health, Fudan University, Shanghai, China; ^3^National Clinical Research Center for Aging and Medicine, Huashan Hospital, Fudan University, Shanghai, China; ^4^Institute of Science and Technology for Brain-Inspired Intelligence, Fudan University, Shanghai, China; ^5^CAS Center for Excellence in Brain Science and Intelligence Technology, Shanghai, China; ^6^Department of The State Key Laboratory of Medical Neurobiology and MOE Frontiers Center for Brain Science, Institutes of Brain Science, Fudan University, Shanghai, China

**Keywords:** idiopathic normal pressure hydrocephalus (INPH), white matter microstructure, motor pathways, sensory pathways, gait indices

## Abstract

**Background**: Idiopathic normal pressure hydrocephalus (iNPH) is a common disease in elderly adults. Patients with iNPH are generally characterized by progressive gait impairment, cognitive deficits, and urinary urgency and/or incontinence. A number of radiographic studies have shown that iNPH patients have enlarged ventricles and altered brain morphology; however, few studies have focused on the relationships between altered brain structure and gait dysfunction due to iNPH. Thus, this study aimed to evaluate the abnormalities of white matter (WM) correlated with gait impairment in iNPH patients and to gain a better understanding of its underlying pathology.

**Methods**: Fifteen iNPH patients (five women, 10 men) were enrolled in this study, and each patient’s demographic and gait indices were collected. First, we performed a correlation analysis between the demographic and gait indices. Then, all gait indices were grouped according to the number of WM hyperintensities (WMH) among each WM tract (JHU WM tractography atlas), to perform comparative analysis.

**Results**: Considering sex and illness duration as covariates, correlation analysis showed a significantly negative correlation between step length (*r* = −0.80, *p* = 0.001), pace (*r* = −0.84, *p* = 2.96e-4), and age. After removing the effects of age, sex, and illness duration, correlation analysis showed negative correlation between step length (*r* = −0.73, *p* = 0.007), pace (*r* = −0.74, *p* = 0.005), and clinical-grade score and positive correlation between 3-m round trip time (*r* = 0.66, *p* = 0.021), rising time (*r* = 0.76, *p* = 0.004), and clinical-grade score. Based on WMH of each white matter tract, gait indices showed significant differences (*p* < 0.05/48, corrected by Bonferroni) between fewer WMH patients and more WMH in the middle cerebellar peduncle, left medial lemniscus, left posterior limb of the internal capsule (IC), and right posterior limb of the IC.

**Conclusions**: Our results indicated that iNPH patients exhibited gait-related WM abnormalities located in motor and sensory pathways around the ventricle, which is beneficial to understand the underlying pathology of iNPH.

## Introduction

Idiopathic normal pressure hydrocephalus (iNPH) is a common reversible syndrome characterized by progressive gait impairment, cognitive deficits, urinary urgency and/or incontinence, ventricular enlargement, and normal mean intracranial pressure, which typically occurs in the elderly (>60 years; Adams et al., 1965; Hebb and Cusimano, 2001). Zaccaria et al. (2019) reviewed and demonstrated that the incidence of iNPH was 1.8/100,000–7.3/100,000 per year, without sex differences. Generally, cerebrospinal fluid (CSF) shunting and drainage aid in improving or reversing symptoms of iNPH in the early period (Mirzayan et al., 2010); therefore, accurate diagnosis and appropriate treatment of early iNPH is important. However, the potential pathological mechanism of iNPH remains unclear. Dilated ventricles are the most typical but non-specific clinical-pathological features of iNPH, which are difficult to distinguish from aging, Alzheimer’s disease (AD; El Sankari et al., 2011), and Parkinson’s disease (Morishita et al., 2010). According to the guidelines for the management of iNPH, the tap test is effective in detecting abnormal CSF hydrodynamics and predicting the effectiveness of ventriculoperitoneal shunting (Marmarou et al., 2005; Ishikawa et al., 2008; Mori et al., 2012); however, it has some potential risk as an invasive procedure. Thus, in-depth pathological investigations and identification of specific biomarkers of iNPH are important for accurate diagnosis and treatment.

Magnetic resonance imaging (MRI) has been used as a non-invasive and non-radiative procedure in many disease studies. MRI is used to detect brain structure changes for auxiliary diagnosis by extracting features such as Evans’ index (Brix et al., 2017) and callosal angles (Grahnke et al., 2018). The application of various MRI techniques provided more insights into the etiology and pathology of iNPH and predicted the effectiveness of ventriculoperitoneal shunting. Magnetic resonance elastography is a developing non-invasive imaging method that assesses brain tissue properties such as parenchymal stiffness by propagating acoustic strain waves and quantitatively mapping physical responses (Kruse et al., 2008). Avital et al. found significantly altered stiffness in patients with iNPH in the cerebrum compared with healthy controls, which implies that brain compliance changes and speculated stiffness may be a feature of surgical effects (Perry et al., 2017). Phase-contrast MRI also has been applied for the evaluation of complex CSF dynamics in iNPH (Takizawa et al., 2017; Lindstrom et al., 2018; Tsai et al., 2018; Yamada et al., 2020). Yamada et al. revealed higher stroke volumes, reversed flow rates, and shear stress at the cerebral aqueduct in patients with iNPH compared with healthy controls (Yamada et al., 2020). Takizawa et al. found different pressure gradients and greater rotation in the cerebral aqueduct between patients with iNPH and healthy controls (Takizawa et al., 2017). Lindstrom et al. observed reversal of CSF net flow direction in patients with iNPH (Lindstrom et al., 2018). Consistent with previous studies (Baledent et al., 2004), all results suggested that abnormal CSF dynamics occurred in iNPH, which may be attributed to altered intracranial compliance and absorption obstacles of CSF. Arterial spin-labeling is a quantitative measurement of regional cerebral blood flow (CBF) using blood as an endogenous contrast agent (Wu et al., 2007). Virhammar et al. (2017) reported that reduced perfusion was mainly located in the periventricular white matter (WM) of patients with iNPH compared with healthy controls, which indicates that more focal ischemia may occur in such patients. Thus, we postulated that abnormal CSF dynamics and CBF mode are related to dysfunctional metabolism and obstacles in the transport of neurotoxic substances, which have been reported in some glymphatic MRI studies (Ringstad et al., 2017; Reeves et al., 2020). All these characteristics found in MRI may be inducement resulting in dilated ventricles; however, few studies have determined the interactions between existing etiologies, which is important for obtaining a truly pathological mechanism.

Meanwhile, due to the non-specificity of clinical manifestations, most studies evaluating brain-specific changes also aim to assist in the diagnosis of iNPH and differentiation between iNPH and its comorbid and mimic disorders. For example, a systematic review reported that *T*-tau and *p*-tau may differentiate iNPH from AD and Aβ_42_ from HC (Manniche et al., 2019). Diffusion tensor imaging as an MRI technique determining diffusion characteristics of white matter (WM) has been performed in most studies of patients with iNPH (Hattingen et al., 2010; Kanno et al., 2011; Lenfeldt et al., 2011; Hattori et al., 2012; Kang et al., 2016; Kamiya et al., 2017; Saito et al., 2020). Griffa et al. concluded regarding white matter microstructural alteration of the periventricular, frontal, and temporal regions in patients with iNPH (Griffa et al., 2020); however, few studies have investigated gray matter structural changes in such patients. A study on gray matter structural networks indicated larger global network modularity and decreased betweenness of regional networks in iNPH, and the proposed network analysis may be a promising method for further study (Yin et al., 2018). Meanwhile, some resting-state MRI (rs-MRI) studies showed abnormal functional connectivity (FC) mode in iNPH such as decreased default mode network connectivity and its strong correlation with worsening clinical symptoms (Khoo et al., 2016), and distinguished different FC patterns associated with the triad of iNPH, including gait impairment, cognitive deficits, urinary urgency, and/or incontinence (Ogata et al., 2017), suggesting that the global FC mode and large-scale brain network properties may be useful biomarkers for the assessment of iNPH. Among them, gait disturbances are the most common and earliest symptom of iNPH, which may be related to abnormal functional and structural changes and can also be used as a biomarker for diagnosis; however, few studies focusing on assessing structural changes associated with abnormal gait needs further research.

To date, our understanding of iNPH is limited, including its pathology and etiology. Previous studies have emphasized that accurate diagnosis contributes to the effective treatment of iNPH; however, few iNPH-correlated specific changes have been found for diagnosis. With the development of MRI techniques and research related to iNPH, an increasing number of findings have prompted the combination of imaging and clinical features to gain a better understanding and diagnosis of iNPH. Meanwhile, studies on structural networks related to gait are limited. Therefore, in this study, we first collected a series of gait indices and T2-weighted imaging in patients with iNPH. Following this, each gait index was grouped according to the number of white matter hyperintensities (WMH) of each white matter fiber tract to discover which fiber tracts are associated with gait. Then, we built a gait-correlated structural network according to survival WM regions to better understand iNPH itself and auxiliary clinical applications. Finally, a correlation analysis was applied between the gait indices and demographic and clinical data.

## Material and Methods

### Participants

Fifteen patients with probable iNPH (10 men and five women) were recruited in our study. All patients were from the Department of Neurology of Zhongshan Hospital and diagnosed as probable iNPH which is consistent with the Japanese iNPH Guidelines (third edition; Nakajima et al., 2021) more than one symptom in the clinical triad (gait disturbance, cognitive impairment, and urinary incontinence); above-mentioned clinical symptoms cannot be completely explained by other neurological or non-neurological disease; preceding diseases possibly causing ventricular dilation (including subarachnoid hemorrhage, meningitis, head injury, congenital/developmental hydrocephalus, and aqueductal stenosis) are not obvious; CSF pressure of 200 mm H_2_O or less and normal CSF content; improvement of symptoms after CSF tap test. A series of demographic and clinical indices of patients were collected, including age, sex, illness duration, comorbidities, iNPH grading scale (iNPHGS) score (Kubo et al., 2008), and Fazekas score (Fazekas et al., 1987). Patients were initially divided into a absent-to-mild WMH burden group (Fazekas score 0–2) and a moderate-to-severe WMH burden group (Fazekas score 3–6) (Helenius and Henninger, 2015; Patti et al., 2016). The gait of all patients was evaluated using 10 gait indices including 10-m walking time (s), 10-m step counter (steps), step length (m), pace (m/s), step frequency (steps/min), step width (m), turning 180° (steps), time of timed-up-and-go (TUG) test (s), rising time (s), and turning 180° (s). All participants signed the informed consent forms approved by the Zhongshan Ethics Committee.

### MRI Acquisition

MRI was acquired using a 3.0-T UIH uMR770 system at Zhongshan Hospital Fudan University. All participants were instructed to remain still while custom-fit foam pads were placed on either side of their heads for fixation. Fast spin-echo (FSE) pulse sequences were used to gather T2-weighted images. The following parameters were used for the FSE pulse sequences: repetition time, 4,300 ms; echo time, 107 ms; flip angle = 150°, number of slices 21, transverse orientation, the field of view, 384 × 334; slice thickness, 5 mm; and spacing between slices, 6.5 mm.

### Imaging Preprocessing

Image pre-processing includes registration for aligning all images into the same coordinate system, imaging normalization, and white matter hyperintensities extraction. Although the images were acquired during the same session, a certain amount of subject motion and movement was unavoidable between the slides, leading to image misalignment. For each participant, all slides were aligned using a three-dimensional rigid body image registration algorithm proposed by Ashburner et al. (2000). We then applied a non-rigid normalization toolbox and transformed the images into a standard template of the Montreal Neurological Institute (MNI) space (Friston et al., 2007). Finally, we used the Lesion Segmentation Toolbox (Schmidt et al., 2012) to identify white matter hyperactivities (WMH) automatically for each participant, which could be considered as potential lesions (Debette and Markus, 2010). All the preprocessing, results were examined by a trained neurologist blinded one-by-one to assure the imaging quality.

### Statistical Analysis

Continuous variables were presented as the mean ± standard deviation (SD), minimum-maximum, and were analyzed using Student’s t-test. Pearson correlation coefficients were used to extract the associations between different variables. In addition, voxel-based lesion-symptom mapping (VLSM; Bates et al., 2003) was used to analyze the relationship between WMH and gait indices on a voxel-by-voxel basis, which could help to identify key brain areas of gait function. Different from the previous regular grouping method, VLSM is performed to compare the gait performance in a group of iNPH patients with a common area of WMH with others without WMH in this area. Analysis of covariance was performed to reduce the interferences of age, sex, illness duration, and iNPHGS on the gait parameters. We applied Fisher’s method (Fisher’s combined probability test) to combine the results of statistical tests of various gait indices. Statistical analysis was performed using the MATLAB software, version 2019b (MathWorks Inc.).

## Results

### Correlation With Behavioral Performance

**Figure 1:**
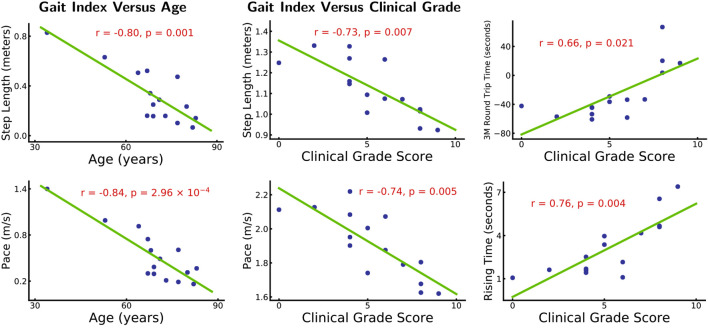
Gait index vs. age and clinical grade. *Left panel: patients’ step length and pace are significantly correlated with age (*r* = −0.80, *p* = 0.001 and *r* = −0.84, *p* = 2.96 × 10^−4^). Other gait indices do not show significant (*p* > 0.05) correlations with age. The effects of sex and illness duration are removed. *Right panel: patients’ step length, pace, 3 m round trip time, and rising time are significantly correlated with their clinical-grade scores (*r* = −0.73, *p* = 0.007, *r* = −0.74, *p* = 0.005 and *r* = 0.66, *p* = 0.021, *r* = 0.76, *p* = 0.004). Other gait indices are not significantly correlated with the clinical grade scores (*p* > 0.05). Age, sex, and illness duration effects are removed.

The demographic and clinical data presented in Table 1, including age, sex, illness duration, iNPHGS score, Fazekas score, comorbidities, and 10 gait indices. The average Fazekas score and iNPHGS score of patients with moderate-to-severe WMH burden were higher than those with absent-to-mild WMH burden. There were no significant differences between the two groups in age, sex, illness duration, comorbidities, and gait indices. All patients recruited in our study had gait improvement after the CSF tap test. Eight of them underwent ventriculo-peritoneal shunt surgery, and all responded well to shunt surgery. The other seven patients refused to undergo shunt due to concerns and hesitations about the risks of surgical treatment. Figure 1 shows the correlation analysis results between age, iNPHGS, and gait indices through a scatter diagram. Choosing sex and illness duration as covariates, correlation analysis showed a significantly negative correlation between step length (*r* = −0.80, *p* = 0.001), pace (*r* = −0.84, *p* = 2.96e-4), and age. Removing effects of age, sex, and illness duration, correlation analysis showed negative correlation between step length (*r* = −0.73, *p* = 0.007), pace (*r* = −0.74, *p* = 0.005), and iNPHGS and positive correlation between time of TUG test (*r* = 0.66, *p* = 0.021), rising time (*r* = 0.76, *p* = 0.004), and iNPHGS.

**Table 1:** Demographic, clinical and gait data of patients.

iNPH patients	Total	Absent-to-mild WMH burden	Moderate-to-severe WMH burden	*t* or *χ*^2^	*p*
*N*	15	6	9	
Age (years)	68.93 ± 12.40	65.83 ± 16.70	71.00 ± 9.70	0.78	0.45
Sex (female, ratio)	4/15	3/6	1/9	2.78	0.10
Illness duration (years)	1.93 ± 1.64	1.50 ± 1.60	2.21 ± 1.70	0.81	0.43
iNPHGS score(points)	5.33 ± 2.47	3.67 ± 2.66	6.44 ± 1.67	2.50	0.03
Fazekas score(points)	2.87 ± 1.52	1.33 ± 0.52	3.89 ± 0.93	6.10	<0.01
Comorbidity			
Hypertension (ratio)	8/15	4/6	4/9	0.71	0.40
Type 2 diabetes (ratio)	6/15	3/6	3/9	0.42	0.52
Current smoking or drinking (ratio)	1/15	0/6	1/9	0.71	0.40
Gait temporal-spatial parameters					
10-m walking time (seconds)	45.24 ± 47.18	25.87 ± 19.83	58.16 ± 56.29	1.33	0.21
10-m step counter (steps)	63.2 ± 60.02	31.83 ± 21.25	84.11 ± 69.23	1.78	0.10
Step length (meters)	0.30 ± 0.23	0.44 ± 0.25	0.21 ± 0.18	2.07	0.06
Pace (m/s)	0.45 ± 0.36	0.63 ± 0.43	0.33 ± 0.26	1.65	0.12
Step frequency (steps/min)	91.05 ± 27.83	81.04 ± 15.31	97.72 ± 32.92	1.15	0.27
Step width (meters)	0.20 ± 0.05	0.21 ± 0.07	0.20 ± 0.04	0.35	0.73
Turning 180 degrees (steps)	9.13 ± 7.95	4.67 ± 2.07	12.11 ± 9.10	1.95	0.07
TUG time (seconds)	45.72 ± 40.82	26.95 ± 19.31	58.23 ± 47.35	1.52	0.15
Rising time (seconds)	3.48 ± 2.24	2.27 ± 1.05	4.29 ± 2.50	1.86	0.09
Turning 180 degrees (time, seconds)	5.66 ± 5.71	3.17 ± 2.14	7.32 ± 6.82	1.43	0.18

*All the indices are shown as mean ± standard deviation (SD). iNPH, idiopathic normal pressure hydrocephalus. WMH, white matter hyperintensity. iNPHGS, iNPH grading scale*.

### Correlation Between WM-Tract Damage and Gait Indices

**Figure 2:**
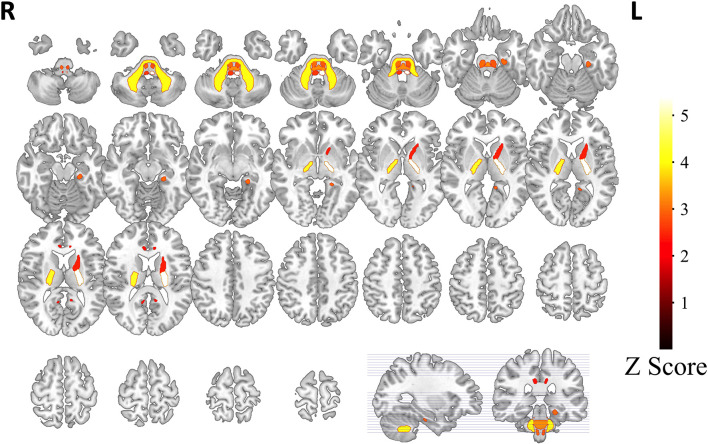
White matter tracts related with gait indices. Based on white matter hyperintensities extracted from every white matter tract, patients are separated into two groups. White matter tracts with significant gait index differences (indicated as *z*-scores in the graph) in the two groups are represented. Tracts with a hotter color have stronger relationships with gait indices.

**Figure 3:**
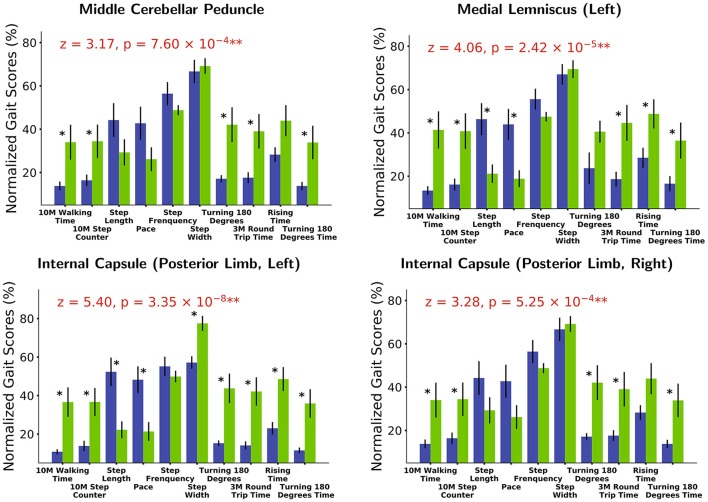
White matter tracts with strongest gait index relationships. Based on white matter hyperintensities (WMH) extracted from different white matter tracts, patients are separated into two groups (blue in the graph: patients with fewer WMHs. Green: those with more WMHs). Various gait score (normalized based on maximum values) differences are observed between the two groups. Gait score differences are combined (Fisher’s method) to extract white matter tracts most strongly related with gait indices [middle cerebellar peduncle (MCP): *z* = 3.17, *p* = 7.60 × 10^−4^ and medial lemniscus (left): *z* = 4.06, *p* = 2.42 × 10^−5^; internal capsule (IC; posterior limb, left): *z* = 5.40, *p* = 3.35 × 10^−8^ and internal capsule (posterior limb, right): *z* = 3.28, *p* = 5.25 × 10^−4^]. *p < 0.1, **p < 0.001.

Based on white matter hyperintensities of each white matter tract, gait indices were significantly different (*p* < 0.05) between fewer WMH patients and more WMH in the middle cerebellar peduncle (MCP), pontine crossing tract (a part of MCP), left corticospinal tract (CST), right corticospinal tract, left medial lemniscus, right medial lemniscus, left anterior limb of the internal capsule (IC), left posterior limb of the internal capsule, right posterior limb of the internal capsule, left cingulum (cingulate gyrus), right cingulum (cingulate gyrus), and left cingulum (hippocampus). After correction by Bonferroni correction, significant correlations (*p* < 0.05/48), four white matter tracts survived, including the MCP, left medial lemniscus, left posterior limb of the internal capsule, and right posterior limb of the internal capsule. The spatial location of white matter tracts related to gait indices is shown in Figure 2, Table 2, and the differences in the gait indices of the four survival white matter tracts are shown in Figure 3.

**Table 2:** Montreal Neurological Institute (MNI) coordinates of peak voxels for white matter tracts.

Fiber tracts	X	Y	Z	Fisher’s method *z*	*p*
Middle cerebellar peduncle	8	−20	−32	7.94	9.99 × 10^−16^
Medial lemniscus (left)	−8	−38	−35	6.83	4.20 × 10^−12^
Internal capsule (posterior limb, left)	−12	−3	5	5.43	2.79 × 10^−8^
Internal capsule (posterior limb, right)	9	−3	3	6.39	8.56 × 10^−11^

*X, Y, and Z refer to MNI coordinates, and Fisher’s method *z* and *p* refer to the *z* and *p* values of the peak voxels*.

## Discussion

In our study, we focused on gait-related WM tract damage in iNPH based on the brain atlas. This study performed a new computer-based automated method for WMH detection based on lesion segmentation and VLSM, which might be a promising supplementary analysis protocol for T2-weighted imaging in the future. Besides, this study is a new attempt to combine MRI data and clinical behavior data in iNPH, which has been rare in the past. Our results provide evidence that there are local brain structural changes associated with gait in iNPH. We found a significant correlation between gait indices and age. After removing the effects of age, sex, and illness duration, correlation analysis showed a significant correlation between gait indices and clinical-grade scores. In grouping based on the number of abnormal WMH, all gait indicators also showed significant differences in regional WM tracts, including the MCP, bilateral medial lemniscus, bilateral posterior limb of the internal capsule, bilateral CST, left anterior limb of the internal capsule, and cingulum. We believe that damage to the brain structure may play an important role in gait abnormalities in patients with iNPH. Beyond the traditional view on elderly-onset iNPH, a 34-year-old young woman was recruited in our study, who met all criteria of probable iNPH, and no evidence of other diseases possibly causing ventricular dilation was found. She was diagnosed as probable iNPH according to the latest version of guidelines (Nakajima et al., 2021), which removed the age limit of “onset at 60 s or older” in old version guideline (Mori et al., 2012).

Generally, abnormal WMH on T2-weighted imaging represents some abnormal WM structure changes, including neural degeneration, ischemia, myelin impairments, and edema. Consistent with numerous previous results, many altered white matter microstructures around the ventricles were also detected in our iNPH participants; however, the underlying pathological mechanisms are unclear. Most studies based on diffusion-weighted imaging showed WM microstructural alteration of the periventricular in patients with iNPH by using fractional anisotropy (FA), mean diffusivity (MD), axial diffusivity (AD), and radial diffusivity (RD), which are sensitive to changes in the WM microstructure. For example, compared with healthy controls, patients with iNPH showed increased FA, MD, and AD in the periventricular section of the CST (Hattingen et al., 2010; Hattori et al., 2012; Saito et al., 2020); decreased FA; and increased RD in the upper section of the CST and corpus callosum (Kanno et al., 2011; Saito et al., 2020), which may result from the mechanical force exerted by the abnormal ventriculomegaly on the surrounding tissues. Aso et al. suggested that one of the mechanisms of ventricular enlargement is that changes in the venous drainage pattern could result in CSF absorption disorder in patients with iNPH (Aso et al., 2020). Kamiya et al. (2017) reported an increased orientational coherence at the CST in patients with iNPH after CSF shunt surgery, which supports the oppression of periventricular tissue. Meanwhile, a review also concluded that chronic cycle disturbance in the CSF may cause periventricular edema and eventually lead to local ischemia (Brautigam et al., 2019), as reported by previous research (Virhammar et al., 2017). Thus, we speculate that the microstructural alteration of periventricular WM may play a role in the pathological mechanism of hydrocephalus, which requires further study. The reason why we performed T2-weighted imaging analysis to show WM tract damage was that many iNPH patients couldn’t tolerate a longer MRI scan time due to their cognitive impairment and urinary incontinence. Previous studies have demonstrated that WMH on the T2-weighted imaging was associated with an increased risk of stroke, dementia, and death (Debette and Markus, 2010). Different from the traditional visual semiquantitative scales (such as the Fazekas scale) or manual volumetric measurements, our study performed an automated algorithm to segment WMH lesions. We were more concerned about the locations and functional roles of WM tract damage than the volumes. VLSM investigates the relationship between a common area and a particular behavior by comparing the performance of individuals with and without the lesion on a voxelwise basis. Using VLSM is more helpful to understand whether WMH in a specific area disrupts the performance of a specific behavior. VLSM has been initially applied in exploring the relationship between WMH location and poor executive function, mental processing speed, memory, and verbal abilities in the previous studies (Smith et al., 2011; Ramirez et al., 2014; Camerino et al., 2021). But there is a rare study to discuss the relationship between gait performance and WMH burden. How to distinguish non-ischemic WMH caused by CSF extravasation from ischemic WMH is still somewhat controversial, and there is no objective, unambiguous and non-disputable consensus currently (Kim et al., 2008). Previous studies have shown that hypertension and diabetes mellitus may correlate with whatever periventricular WMH or deep WMH (King et al., 2014; de Bresser et al., 2018; Wang et al., 2020). Due to the small sample size, we were unable to perform a stratified analysis of vascular factors. We cannot deny that comorbidities in elderly iNPH patients such as hypertension and diabetes mellitus may cause some WMH load in our study. Nevertheless, the purpose of our study was to explore the relationship between WMH location and gait performance. As Finsterwalder et al.’s report, CADASIL patients showed only mild gait impairment in the rhythm domain despite severe WMH burden, which indicated that the gait impairment caused by pure vascular-original WMH was relatively minor especially combined with a definitively diagnosed gait disorder (Finsterwalder et al., 2019). Thus, we consider that the comorbidities will not affect our results.

Previous studies have shown abnormal gait in patients with iNPH, including shortened stride length, difficulties in turning, and difficulty in balance (Hebb and Cusimano, 2001; Marmarou et al., 2005; Ishikawa et al., 2008; Mori et al., 2012). In our study, we found that step length and pace were significantly negatively correlated with age, without the influence of sex and illness duration. We also found a significant negative correlation between clinical grade score and step length and pace and a significant positive correlation between clinical grade score and 3 m round trip time and rising time, removing the effects of age, sex, and illness duration. Clinical grade scores are evaluated according to the severity of clinical symptoms; the higher the score, the more severe the illness (Kubo et al., 2008). These findings suggest that changes in gait in patients with iNPH may be influenced by both aging and iNPH. Satow et al. (2017) showed that extended drainage times with age were mainly distributed in the periventricular region in healthy controls, which implied altered venous drainage. Thus, we speculated that periventricular structural and dynamic changes may be associated with gait disturbances.

Comparative analysis in our study reported that patients with iNPH with more WMH had poor gait performance, with the fiber tracts most significantly correlated with gait indices: MCP, left medial lemniscus, and bilateral posterior limb of the IC; the fiber tracts that were significantly associated with gait were the bilateral CST (section of the pons), right medial lemniscus, left anterior limb of the IC, and cingulum. MCP is the biggest peduncle of the three cerebellar peduncles, which arise from the pontine nucleus to the contralateral cerebellar cortex. As the main cerebellar afferent fiber, the MCP receives information from the cerebral cortex. The medial lemniscus is the proprioceptive conduction pathway of the limbs, whose structure is through the medulla oblongata, pons, and midbrain to thalamus and projecting proprioception by the posterior limb of IC to the contralateral sensorimotor cortex (Navarro-Orozco and Bollu, 2020). The IC is the concentration area of projecting fibers that connect the cerebral cortex to the thalamus, brainstem, and spinal cord, in which minor lesions could also cause sensorimotor disorders (Emos and Agarwal, 2020). Kanno et al. reported the number of steps of the Timed “Up and Go” test was negatively correlated with FA in the left supplementary motor area and left anterior limb of IC (Kanno et al., 2011). The structure of the CST is the largest descending fiber tract in the spinal cord, which originates from the primary motor cortex and premotor areas as the major motor pathway that innervates the lower motor neurons (Jang, 2014; Rong et al., 2014). Hattingen et al. (2010) also found that MD in the CST was correlated with severe gait disturbances. The cingulum is an association fiber whose function is to connect the neocortex to the limbic system, and its structural damage may also result in an information transmission barrier. According to our results, we speculated that WM microstructural damage in the above may affect pathways correlated with motor function, consistent with previous research. Kang et al. (2016) reported decreased FA in the MCP of patients with iNPH compared with HC. An animal model of hydrocephalus found distinct cerebellar changes in biochemical parameters (Kondziella et al., 2002). The cerebellum is involved in motor control through two main pathways: the efferent pathway (cerebello-thalamocortical) and the afferent pathway (cortico-pontocerebellar; Palesi et al., 2017). Meanwhile, Griffa et al. indicated that frontoparietal-subcortical-cerebellar circuits may be a vulnerable area for the pathophysiological mechanisms of iNPH (Griffa et al., 2020). Therefore, in our study, we believe that WM microstructural alterations of the periventricular may induce disturbance transmission of information correlated with abnormal gait.

In conclusion, we found that WM microstructural alterations of periventricular in patients with iNPH in our study and some of the WM damage were associated with gait indices and were mainly located in some neuroanatomical circuits. We also found a significant correlation between gait indices, age, and grade score. These results suggest that abnormal gait in patients with iNPH may be related to both aging and pathological changes. Meanwhile, gait-related WM microstructural damage was located in the motor and sensory pathways, suggesting an information transmission barrier in such patients.

## Limitations

While our research reveals that patients with iNPH exhibit dysfunctions in some neuroanatomical circuits, our study also has several limitations. First, our sample size was relatively limited due to the low incidence of iNPH and traumatic diagnostic tests. Second, in the present study, we used T2-weighted imaging, which has a lower spatial resolution, to analyze WM damage. Besides, many patients with iNPH are elderly-onset with metabolic comorbidities. It is difficult to remove WMH of presumed vascular origin during imaging analysis. In future studies, we should perform a large-sample longitudinal cohort study of iNPH to better understand iNPH-related short-and medium–term plasticity mechanisms through stratification of vascular factors and follow-up of the dynamic changes of brain structure and function. Furthermore, we can also explain the structural and functional changes of iNPH-related WMH combined with advanced white matter imaging technologies. Additionally, the association between multiple clinical manifestations in patients with iNPH, such as gait and cognition, should also be considered in further research to help us understand its pathology.

## Data Availability Statement

The datasets presented in this article are not readily available because of the Subject’s personal privacy protection. Requests to access the datasets should be directed to He Wang, hewang@fudan.edu.cn; Jing Ding, ding.jing@zs-hospital.sh.cn.

## Ethics Statement

The studies involving human participants were reviewed and approved by Zhongshan Ethics Committee. The patients/participants provided their written informed consent to participate in this study. Written informed consent was obtained from the individual(s) for the publication of any potentially identifiable images or data included in this article.

## Author Contributions

Y-mT, YY, SX, XL, FH, HW, JD, and XW designed the study. Y-mT, YY, SX, and XL collected and analyzed data. Y-mT and YY drafted the manuscript. FH, HW, JD, and XW contributed to critical review and revision of the report. All authors contributed to the article and approved the submitted version.

## Conflict of Interest

The authors declare that the research was conducted in the absence of any commercial or financial relationships that could be construed as a potential conflict of interest.

## Publisher’s Note

All claims expressed in this article are solely those of the authors and do not necessarily represent those of their affiliated organizations, or those of the publisher, the editors and the reviewers. Any product that may be evaluated in this article, or claim that may be made by its manufacturer, is not guaranteed or endorsed by the publisher.
